# Aluminum Enhances Growth and Sugar Concentration, Alters Macronutrient Status and Regulates the Expression of *NAC* Transcription Factors in Rice

**DOI:** 10.3389/fpls.2017.00073

**Published:** 2017-02-14

**Authors:** Marcos Moreno-Alvarado, Soledad García-Morales, Libia Iris Trejo-Téllez, Juan Valente Hidalgo-Contreras, Fernando Carlos Gómez-Merino

**Affiliations:** ^1^Biotechnology, Colegio de Postgraduados Campus CórdobaAmatlán de los Reyes, Mexico; ^2^Plant Biotechnology, CONACYT-CIATEJ, El Bajío del ArenalZapopan, Mexico; ^3^Soil Science–Plant Nutrition, Colegio de Postgraduados Campus MontecilloMontecillo, Mexico

**Keywords:** *Oryza sativa*, beneficial elements, aluminum, amino acid, nutrient concentration, NAM subfamily, qRT-PCR

## Abstract

Aluminum (Al) is a beneficial element for some plant species, especially when used at low concentrations. Though some transcription factors are induced by exposure to this element, no data indicate that Al regulates the expression of *NAC* genes in rice. In this study we tested the effect of applying 200 μM Al on growth, chlorophyll, amino acids, sugars, macronutrient concentration and regulation of *NAC* transcription factors gene expression in 24-day-old plants of four rice (*Oryza sativa* ssp. indica) cultivars: Cotaxtla, Tres Ríos, Huimanguillo and Temporalero, grown hydroponically under greenhouse conditions. Twenty days after treatment, we observed that Al enhanced growth in the four cultivars studied. On average, plants grown in the presence of Al produced 140% more root dry biomass and were 30% taller than control plants. Cotaxtla and Temporalero showed double the root length, while Huimanguillo and Cotaxtla had three times more root fresh biomass and 2.5 times more root dry biomass. Huimanguillo plants showed 1.5 times more shoot height, while Cotaxtla had almost double the root dry biomass. With the exception of Tres Ríos, the rest of the cultivars had almost double the chlorophyll concentration when treated with Al, whereas amino acid and proline concentrations were not affected by Al. Sugar concentration was also increased in plants treated with Al, almost 11-fold in comparison to the control. Furthermore, we observed a synergic response of Al application on P and K concentration in roots, and on Mg concentration in shoots. Twenty-four hours after Al treatment, *NAC* transcription factors gene expression was measured in roots by quantitative RT-PCR. Of the 57 *NA*C transcription factors genes primer-pairs tested, we could distinguish that 44% (25 genes) showed different expression patterns among rice cultivars, with most of the genes induced in Cotaxtla and Temporalero plants. Of the 25 transcription factors up-regulated, those showing differential expression mostly belonged to the NAM subfamily (56%). We conclude that Al improves growth, increases sugar concentration, P and K concentrations in roots, and Mg concentration in shoots, and report, for the first time, that Al differentially regulates the expression of *NAC* transcription factors in rice.

## Introduction

Aluminum comprises approximately 7% of the Earth's crust, making it the third most abundant element (after oxygen and silicon) and the most abundant metal on Earth (Matsumoto and Motada, 2012; Matsumoto et al., 2015). Aluminum, which occurs naturally as a free metal, is so chemically reactive that native specimens are rare and limited to reducing environments. Its biological functions are complex and have been largely associated with physiological disorders in plants (Matsumoto and Motada, 2012). Indeed, Al is a major growth-limiting factor in acid soil. It is estimated that approximately 30–40% of arable land and up to 70% of the world's potentially arable land is occupied by acid soils. In these soils, Al is solubilized into ionic forms, especially when the soil pH falls to lower than 5. Under such conditions, most Al exists as the octahedral hexahydrate, $\text{Al}{\left({\text{H}}_{2}\text{O}\right)}_{6}^{3+}$, often abbreviated as Al^3+^, which is believed to be the most toxic Al form (Kochian et al., 2005; Ma and Ryan, 2010). Sade et al. (2016) have recently reviewed toxicity and tolerance of Al in plants. Nevertheless, Al has also been referred to as a beneficial element. Especially in plants native to tropical regions where acid soils are common, Al stimulates plant growth and enhances P uptake (Osaki et al., 1997). In tea (*Camellia sinensis*) and Indian rhododendron (*Melastoma malabathricum*), Al induces plant growth, activates antioxidant responses and improves nutrient status (Ghanati et al., 2005; Watanabe et al., 2005). In alfalfa (*Medicago sativa*), Al enhances root growth (Zhang et al., 2007), whereas in common bean (*Phaseolus vulgaris*) it improves root and shoot growth as well as antioxidant activity (Du et al., 2010) and in maize (*Zea mays*) it stimulates leaf growth (Wang et al., 2015). In rice (*Oryza sativa*), Al stimulates growth (Osaki et al., 1997), root elongation (Famoso et al., 2011), shoot height and chlorophylls as well as carotenoids concentrations (Nhan and Hai, 2013).

By definition, plants that accumulate >1 mg g^−1^ Al (in dry biomass weight) are considered Al-hyperaccumulators (Jansen et al., 2002). These plants are able to use Al to stimulate growth and trigger mechanisms against herbivores, as occurs with tall fescue (*Festuca arundinacea*) (Potter et al., 1996). A possible explanation for this defense response in tall fescue is that Al deposits form an olfactory or tactile barrier, preventing female insects from laying their eggs, which might account for lower numbers of grubs in treated plots (Potter et al., 1996).

According to Pilon-Smits et al. (2009), the beneficial effects of Al in plants are associated with the promotion of growth, activation of antioxidant mechanisms, and increased P availability and decreased Fe toxicity. Consequently, Al may be used as a biostimulant to promote growth and productivity in crop plants, especially when used at low concentrations. Nevertheless, studies on the beneficial effects of Al on plant metabolism are relatively scarce, in comparison to those related to the toxic effects and tolerance mechanisms of plants exposed to this metal (Hajiboland et al., 2013a).

According to recent reports, increasing evidence points to an important role played by transcription factors in Al signal perception and transduction (Yokosho and Ma, 2015). The C2H2-type zinc-finger transcription factor *STOP1* (sensitive to proton rhizotoxicity 1) is not sensitive to either to Al or low pH (Liu et al., 2009; Sawaki et al., 2009). *STOP2*, a homolog of *STOP1* in Arabidopsis, is regulated by the STOP1 protein (Kobayashi et al., 2014) in response to acidic media and Al. The gene *ART1* (*Al resistance transcription factor 1*) is another C2H2-type zinc-finger transcription factor found in rice (Yamaji et al., 2009) that regulates the expression of at least 31 genes such as *STAR1, STAR2, Nrat1, OsALS1*, and *OsMGT1*, which are involved in Al transport. In addition, ART1 activates the transcription of the genes *OsCDT3* and *OsFRDL4* involved in citrate secretion in response to Al (Yokosho and Ma, 2015). The proteins STOP1 and ART1 regulate only two genes in common (*AtMATE*/*OsFRDL4* and *ALS3*/*STAR2*), which may suggest that such proteins are involved in different Al-tolerance pathways. The WRKY46 transcription factor belongs to the family WRKY, and is a negative regulator of the *AtALMT1* gene, which in turn is a key regulator of Al tolerance in Arabidopsis (Ding et al., 2013). Finally, ASR5 (Abscisic acid, stress and ripening 5) is a transcription factor found in rice, closely related to Al tolerance (Arenhart et al., 2014). Nevertheless, there are no reports on the involvement of NAC transcription factors in plant responses to Al exposure. NAC is an acronym derived from the names of the three genes first described as containing the domain, namely *NAM* (*no apical meristem*), *ATAF1,2* (*Arabidopsis transcription activation factor*), and *CUC2* (*cup-shaped cotyledon*). These proteins are plant-specific transcription factors reported to be involved in developmental and growth processes, as well as in the coordination of responses in plant cells to environmental cues of both a biotic and abiotic nature (Nuruzzaman et al., 2013; Nakashima et al., 2014; Hong et al., 2016).

Rice is one of the most Al-tolerant crop species in the world. It can tolerate two- to five-fold higher Al levels than wheat, sorghum or maize (Famoso et al., 2010; Arenhart et al., 2014). Herein, we determined the effect of Al on plant growth, amino acids, proline, soluble sugars and macronutrients concentrations in roots and shoots of four Mexican rice cultivars (Cotaxtla, Tres Ríos, Huimanguillo, and Temporalero), as well as the expression profiling of *NAC* genes transcription factors in roots. We observed that Al increased root and shoot growth, as well as soluble sugars in leaves and P in roots. Interestingly, we report for the first time, to our knowledge, the induction of *NAC* gene expression in Al-treated rice plants.

## Materials and methods

### Rice cultivars and experimental conditions

We evaluated four Mexican rice cultivars (ssp. indica): Cotaxtla, Tres Ríos, Huimanguillo and Temporalero, provided by the National Rice Germplasm Bank housed in the National Institute for Forestry, Agriculture and Livestock Research (INIFAP) located in Zacatepec, Mexico (18°39′ NL, 99°12′ WL, 910 masl).

For germination experiments, seeds were surface sterilized with 70% ethanol for 7 min, and soaked for 30 min in a solution containing 3% sodium hypochlorite and a drop of Tween-20. Subsequently, seeds were rinsed 5 times with distilled water, dried on filter paper under a fume hood and then sown in 500 mL flasks containing MS medium (Murashige and Skoog, 1962), supplemented with 3% sucrose (w/v) and solidified with 0.8% agar. Flasks were incubated in darkness at 28°C for 72 h. Subsequently, plantlets were grown under a day-length of 12 h at 26/22°C (day/night), 70% humidity and 700 μmol m^−2^ s^−1^ light intensity. Eleven days after germination, plants were transferred to 12 L trays containing Yoshida nutrient solution, which contained 1.43 mM NH_4_NO_3_, 1.00 mM CaCl_2_ 2H_2_O, 1.64 mM MgSO_4_ 7H_2_O, 0.13 mM K_2_SO_4_, 0.32 mM NaH_2_PO_4_.2H_2_O, 1.00 mM Fe-EDTA, 7.99 μM MnCl_2_ 4H_2_O, 0.15 μM ZnSO_4_ 7H_2_O, 0.15 μM CuSO_4_ 5H_2_O, 0.075 μM (NH_4_)_6_Mo_7_O_24_4H_2_O and 1.39 μM H_3_BO_3_ (Yang et al., 1994). The pH in the solution was adjusted to 5.5. Thirteen days after transplanting, the nutrient solution was completely replaced and rice plants were grown under control conditions or subjected to Al treatment (200 μM AlCl_3_ at pH 4.2) for 20 days. The hydroponic solution was replaced every 5 days, and it was not oxygenated since rice can cope with low (hypoxia) or absent oxygen (anoxia) (Yamauchi et al., 2000; Joshi and Kumar, 2012). These experiments were carried out in a greenhouse under the aforementioned environmental conditions.

### Sample collection

In order to determine chlorophyll, amino acids, proline and macronutrients concentrations, plants were harvested 20 days after treatment application. To carry out the expression profiling analyses of *NAC* genes, plants were sampled before treatment application and 24 h after exposure to Al; immediately after sampling, plants were rinsed with distilled water, separated into roots and shoots, frozen in liquid nitrogen and then stored at −80°C until RNA extraction.

### Plant growth and biomass production

Plant growth and biomass production were determined 20 days after treatment applications. Plant height was estimated measuring from the shoot base to the tip of the flag leaf. Root length was measured from the shoot base to the tip of the longest root hair. Dry biomass weight was determined 48 h after drying samples (roots and shoots) at 70°C in a forced-air drying oven (Riossa HCF-125D; Monterrey, N.L., Mexico).

### Quantification of chlorophylls and total free amino acids

Chlorophylls (*a, b*, and total) and total free amino acid concentrations in leaf were determined by ethanolic extraction according to Geiger et al. (1998). We sampled the 2nd and 3rd youngest leaves and samples were immediately frozen in liquid nitrogen and then stored at −80°C, until analyzed. From those samples, 20 mg of fresh tissue were taken and mashed with pestle and mortar in liquid nitrogen. We carried out two extractions with 80% ethanol and a third one with 50% ethanol. During the three extractions, samples were incubated at 80°C for 20 min, and then the three extracts were mixed. For chlorophyll quantification, we took 325 μL of the final extract and mixed it with 850 μL 98% ethanol, and recorded chlorophyll concentrations at 645 and 665 nm. Chlorophyll quantification was calculated using the following formulas (where FBW is fresh biomass weight):

$$\begin{array}{l} \text{Chlorophyll }a\text{ concentration }(\mu \text{g }{\text{mg}}^{-1}\text{ FBW})=(5.46\\ \;\times \text{ Absorbance 665nm})-(2.16\;\times \text{ Absorbance 645nm})\\ \text{Chlorophyll }b\text{ concentration }(\mu \text{g }{\text{mg}}^{-1}\text{ FBW})=(9.67\\ \;\times \text{ Absorbance 645nm})-(3.04\;\times \text{ Absorbance 665nm})\\ \text{Total Chlorophyll concentration }(\mu \text{g }{\text{mg}}^{-1}\text{ FBW})=\\ \text{ chlorophyll }a\;+\text{chlorophyll }b \end{array}$$

We then determined total free amino acid concentrations by the ninhydrin method (Moore and Stein, 1954). We took 250 μL of the final extract, added 250 μL of the sodium citrate [citric acid (16 mM) + sodium citrate (34 mM), pH 5.2] − ascorbic acid (0.2% in sodium citrate solution) buffer solution, and 500 μL ninhydrin (1% in 70% ethanol). Subsequently, samples were incubated in a water bath at 95°C for 20 min. We used leucine (10 mM in 70% ethanol) to construct the standard curve, and calculated the amino acid concentrations at 570 nm absorbance.

### Quantification of free proline and total soluble sugars

Total free proline was determined in rice plant shoots according to Bates et al. (1973). We used 50 mg of previously lyophilized and crushed tissue. Then we carried out a first extraction by macerating the samples with 5 mL 3% sulfosalicylic acid, and filtering the sample with filter paper No. 4. Subsequently, we mixed 2 mL of ninhydrin solution (2.5% w/v contained in the solution of 60% concentrated acetic acid and 40% phosphoric acid 6 M), plus 2 mL concentrated glacial acetic acid and 2 mL of the extract of each sample. The mixture was incubated in a water bath at 95°C for 40 min, and the reaction was stopped by placing samples on ice. After the reaction, 4 mL toluene were added to each sample, mixed briefly (vortex) and incubated at room temperature for 15 min. For proline quantification, we constructed a standard curve using L-proline (400 nM mL^−1^) and the corresponding absorbance was measured at 520 nm. Quantification of total soluble sugars in leaves was determined according to the protocol described by Bailey (1958). Shoot tissues were lyophilized, powdered and weighed. Later, extraction was performed using 50 mL 80% ethanol at constant boiling on a thermal shaker with occasional stirring. The supernatant was filtered and the total volume was gauged to 10 mL using 80% ethanol. One mL of the final extract was taken, placed on a 50 mL glass tube and 5 mL anthrone (0.4% in concentrated sulfuric acid) were added; during the process samples were kept on ice. Afterwards, samples were incubated in a water bath at 95°C for 15 min; the reaction was stopped by placing samples on ice. For soluble sugars quantification, we constructed a standard curve using sucrose (0.015% w/v) and measurements were carried out at an absorbance of 600 nm.

### Nutrient concentrations

Once samples were completely dried, they were ground, weighed and subjected to acidic digestion in a mixture of perchloric and nitric acids, according to the protocol described by Alcántar and Sandoval (1999). To determine concentrations of Al, P, K, Ca, and Mg in plant tissues, extracts were analyzed using an inductively coupled plasma atomic emission spectrometer (ICP-OES) (Agilent ICP-AES 725-ES; Victoria, Australia). Nitrogen concentrations were quantified using the Semimicro-Kjeldahl method as described by Bremner (1996), using a catalytic mixture and adding salicylic acid dissolved in concentrated sulfuric acid for the digestion.

### RNA extraction and cDNA synthesis

RNA extraction was carried out with 50 mg plant tissue, using the SV total RNA Isolation System kit (Promega; Madison, WI, USA), according to the manufacturer's protocol, which includes a DNAse I treatment. RNA concentration was measured in a NanoDrop 2000 UV-Vis spectrometer (Thermo Scientific; Waltham, MA, USA). RNA integrity was assessed by electrophoresis on 1% (w/v) agarose gels. In all of the samples, A_260_/A_280_ relation values were equal or higher than 1.8 and A_260_/A_230_ relations were equal or higher than 2.1, meaning that the RNA had good quality for further analyses.

For reverse transcription we used 3.5–5.0 μg total RNA, using the oligo-dT primer for the cDNA first strand synthesis and the enzyme SuperScript III^TM^ RT (Invitrogen; Carlsbad, CA, USA), in a total reaction volume of 20 μL, according to the manufacturer's protocol.

### Primers for RT-PCR analysis

Primers pairs used in this study were those previously reported by García-Morales et al. (2014) and Caldana et al. (2007). We also tested the expression of the following *NAC* genes: *OsNAC6* (Nakashima et al., 2007), *OsNAC5* (Sperotto et al., 2009), and *OsNAC10* (Jeong et al., 2010). As positive controls, we evaluated the expression of two genes previously reported as Al-responsive: *sensitive to Al rhizotoxicity1* (*STAR1*) and *abscisic acid, stress, and ripening 5* (*ASR5*) (Huang et al., 2009; Arenhart et al., 2016). Additionally, we measured the expression of the following transcription factors involved in various plant responses to environmental stress: *OsDREB1A* (Kim et al., 2010), *OsDREB2A* and *OsDREB2B1* (Matsukura et al., 2010), *TRAB1* (Yang et al., 2011), *OsbZIP72* (Lu et al., 2009), and *OsRAN2* (Zang et al., 2010). (Supplementary Material S1). Housekeeping genes tested in this study were *Actin* (Os03g50890), *Actin 1* (Os05g36290), β*-tubulin* (Os01g59150), and *Elongation factor 1*α (Os30g55270). Gene-stability measure (*M*) of reference genes was determined according to Vandesompele et al. (2002) and we selected the most stable reference gene (with the lowest *M*-value) for the calculation of relative expression of *NAC* genes (Supplementary Material S2).

### Real time RT-PCR

Real time RT-PCR was carried out in an ABI Prism 7900HT (Applied Biosystems; Foster City, CA, USA) sequence detection system, using Power SYBR® Green PCR Master Mix 2X (Life Technologies; Carlsbad, CA, USA), according to the manufacturer's protocol. The final concentration of each primer was 250 nM and 20 ng of cDNA in the final volume of 20 μL were used. The PCR reaction conditions were as follows: 50°C for 2 min, 95°C for 10 min, 40 cycles at 95°C for 15 s and 60°C for 1 min. The dissociation curve was obtained after the cycle of the PCR reaction at 95°C for 15 s followed by a constant increase (2%) between 60and 95°C. For each PCR reaction, a dissociation stage was included in order to readily assess the homogeneity of the PCR products, including the presence of primer–dimers, thereby determining the specificity of the PCR reaction (Schmittgen and Livak, 2008). As reference gene we used *Actin* (Os03g50890) in order to normalize the expression of the analyzed genes. All reactions were performed with three technical replicates.

Relative expression of the genes of interest was calculated using the 2^−ΔΔCt^ method (Schmittgen and Livak, 2008). Accordingly, expression data were normalized by subtracting the mean reference gene C_T_ value from its C_T_ value (ΔC_T_). The Fold Change value was calculated using the expression 2^−ΔΔCt^, where ΔΔC_T_ represents ΔC_T condition of interest_ − ΔC_T control_. Results were transformed to log_2_ scale. In accordance with Le et al. (2011) and García-Morales et al. (2014), and considering the biological significance of the differential expression in this study, we adopted a cut-off value of two-fold when analyzing Al induction or repression. The expression levels were designated as “induced” (Fold Change ≥ +2) or “repressed” (Fold Change (≤ −2) only if such differences met the above criteria and passed the Fisher LSD test (Le et al., 2011; García-Morales et al., 2014).

### Statistical analysis

Results are means ± standard error of at least four independent samples per cultivar and treatment. Data were analyzed using the statistical software SAS (SAS Institute, 2004). We carried out an analysis of variance by multifactorial ANOVA, using treatment and cultivar as independent factors. Means comparison was done using Tukey's test with a significance value of 95% (*P* ≤ 0.05), in order to determine significant differences. In order to obtain mean comparisons among rice cultivars regarding *NAC* gene expression, the Fisher LSD (P ≤ 0.05) test was used.

## Results

### Al enhances root and shoot growth

In a preliminary experiment, we tested the effect of 0, 25, 50, 100, 200, and 400 μM Al on the growth and development of cultivars Cotaxtla, Tres Ríos, Huimanguillo, and Temporalero. The stimulant effect of Al on plant growth was observed in all Al concentrations tested, though negative effects of 400 μM Al on root growth and tiller formation in Cotaxtla and Tres Ríos plants were also detected (Supplementary Material S3). Based on those findings, we decided to perform further analysis by comparing 0 (control) and 200 μM Al. Other studies aimed at detecting toxic effects of Al on plant physiology have tested Al concentrations higher than 200 μM. In rice, Famoso et al. (2010, 2011) evaluated 540 and 1290 μM AlCl_3_, whereas Arenhart et al. (2014) tested 450 μM AlCl_3_. As well, Roselló et al. (2015) applied 500 μM AlCl_3_ in rice, while Cançado et al. (2008) applied up to 283 μM of AlK_3_(SO_4_)_3_ to maize plants.

**Figure 1:**
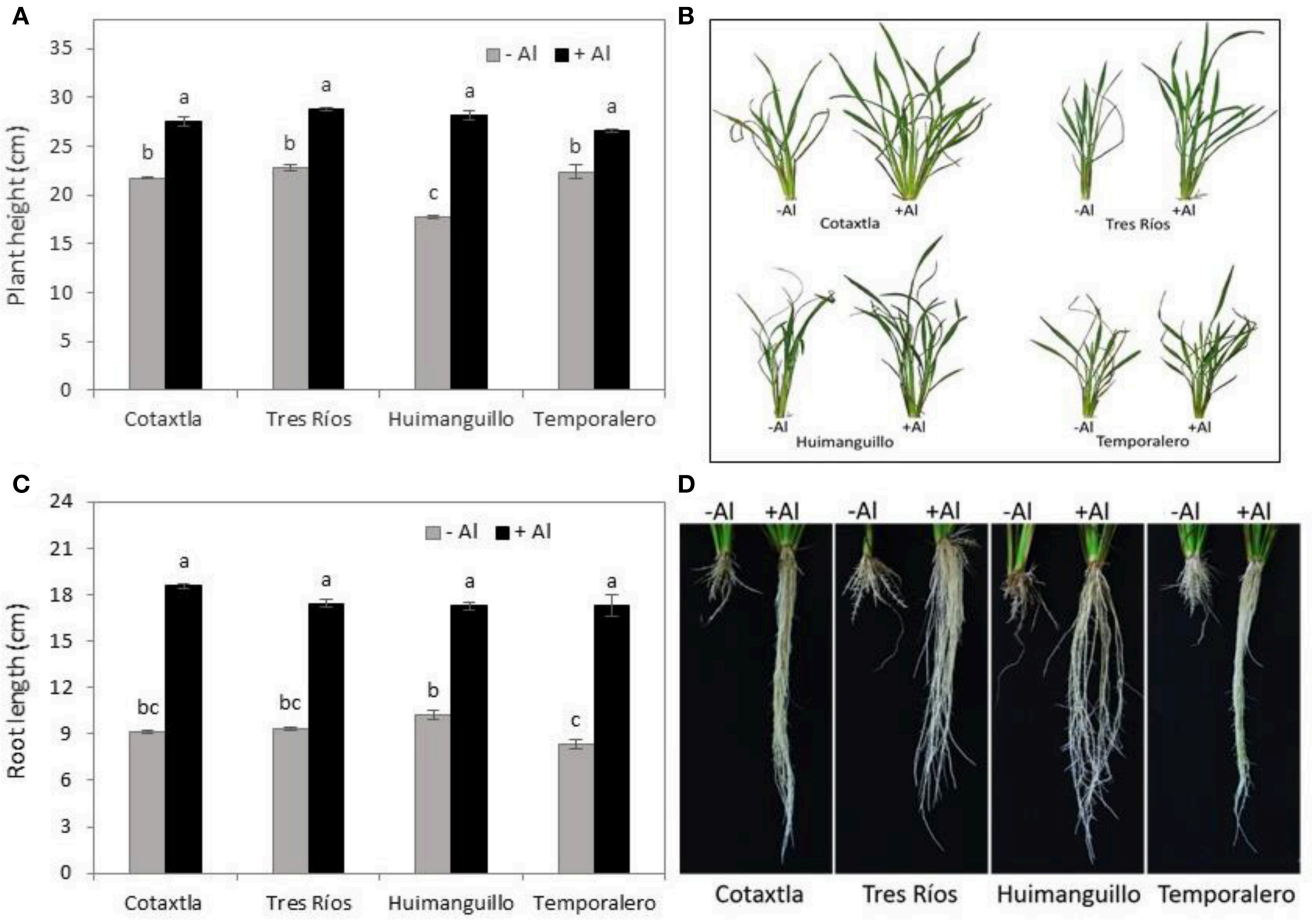
**Growth of rice plants in response to Al treatment**. Plant height **(A)**, root length **(C)** and photographic representation of shoot **(B)** and root **(D)** growth of rice plant cultivars Cotaxtla, Tres Ríos, Huimanguillo and Temporalero grown in the absence (−) or presence of 200 μM Al (+) for 20 days. Values are means ± standard error (SE) from at least five individual plants. Different letters above the column indicate significant differences (Tukey, *P* ≤ 0.05).

In this study, we grew the Mexican rice cultivars Cotaxtla, Tres Ríos, Huimanguillo, and Temporalero hydroponically in Yoshida nutrient solution under greenhouse conditions. Treatments without and with Al (0 and 200 μM AlCl_3_, respectively) were applied to 24-day-old plants for 20 days. We found that plant growth was stimulated by Al (Figure 1). Indeed, plant height increased approximately 30% in Al-treated plants in comparison to control plants. Interestingly, Huimanguillo plants increased 59% in height when treated with the metal in the nutrient solution. Cotaxtla and Tres Ríos showed an increase of 27 and 26%, respectively, while Temporalero displayed the lowest gain, with only 18% in Al-treated plants in comparison to the control. Moreover, plants exposed to Al promoted tillering (Figure 1B).

A more evident beneficial effect of Al was observed in root growth. On average, the four cultivars increased root length by 90% when treated with Al, in comparison with the control. Cotaxtla and Temporalero plants showed almost double the root length, whereas Tres Ríos increased this value by 86% and Huimanguillo by 69% (Figure 1C). Surprisingly, we could observe a higher number of roots in Al-treated plants, in comparison to control plants (Figure 1D).

### Al induces higher fresh and dry biomass production

**Figure 2:**
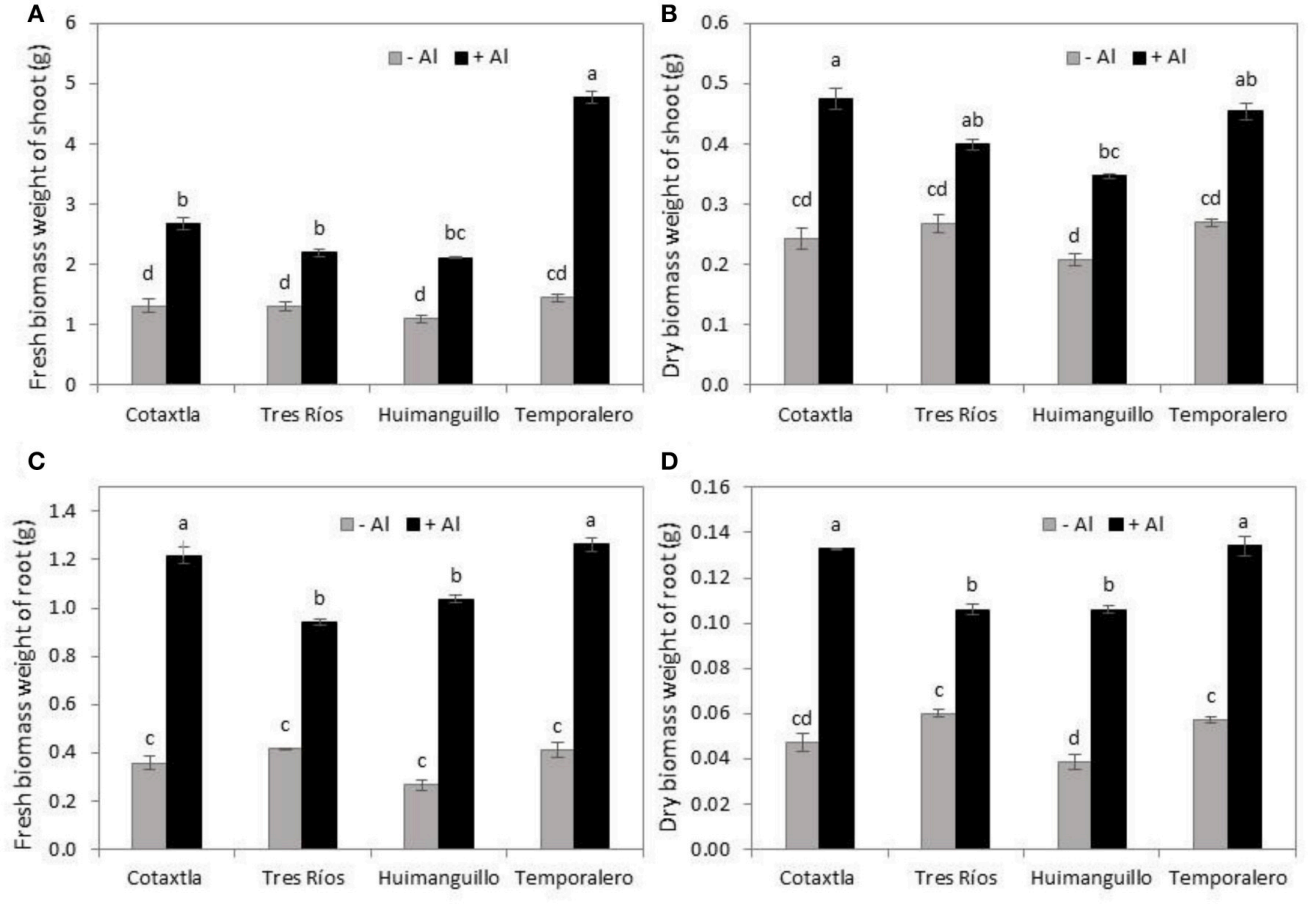
**Fresh and dry biomass production by rice plants in response to Al treatment**. Fresh biomass weight of shoot **(A)** and root **(C)**. Dry biomass weight of shoot **(B)** and root **(D)** of rice plant cultivars Cotaxtla, Tres Ríos, Huimanguillo and Temporalero grown in the absence (−) or presence of 200 μM Al (+) for 20 days. Values are means ± SE from at least five individual plants. Different letters above the column indicate significant differences (Tukey, *P* ≤ 0.05).

Aluminum enhanced biomass production in the four rice cultivars evaluated. In Cotaxtla plants, Al produced double the shoot fresh biomass, whereas it was 1.7 and 1.9 times higher in Tres Ríos and Huimanguillo, respectively; in Temporalero plants it was three times higher, in all cases, in comparison to the control (Figure 2A). A similar trend was observed in shoot dry biomass; consequently, Cotaxtla plants showed double the dry shoot biomass weight, 1.5 higher weights in Tres Ríos, and 1.7 in Huimanguillo and Temporalero, in comparison to control plants. Concerning this variable, we could also observe different responses among cultivars. Cotaxtla plants exhibited the highest biomass production, while the lowest production was recorded in Huimanguillo plants (Figure 2B).

Root fresh biomass weight was also increased by Al treatments; Al-treated Cotaxtla, Humanguillo, and Temporalero plants developed more than three times this weight in comparison to control plants, whereas Tres Ríos showed more than double the value in comparison to the control (Figure 2C). Similar results were observed for root dry biomass weight (Figure 2D), with stronger responses found in Cotaxtla and Temporalero treated with Al.

### Aluminum affects chlorophyll concentrations in rice leaves

**Figure 3:**
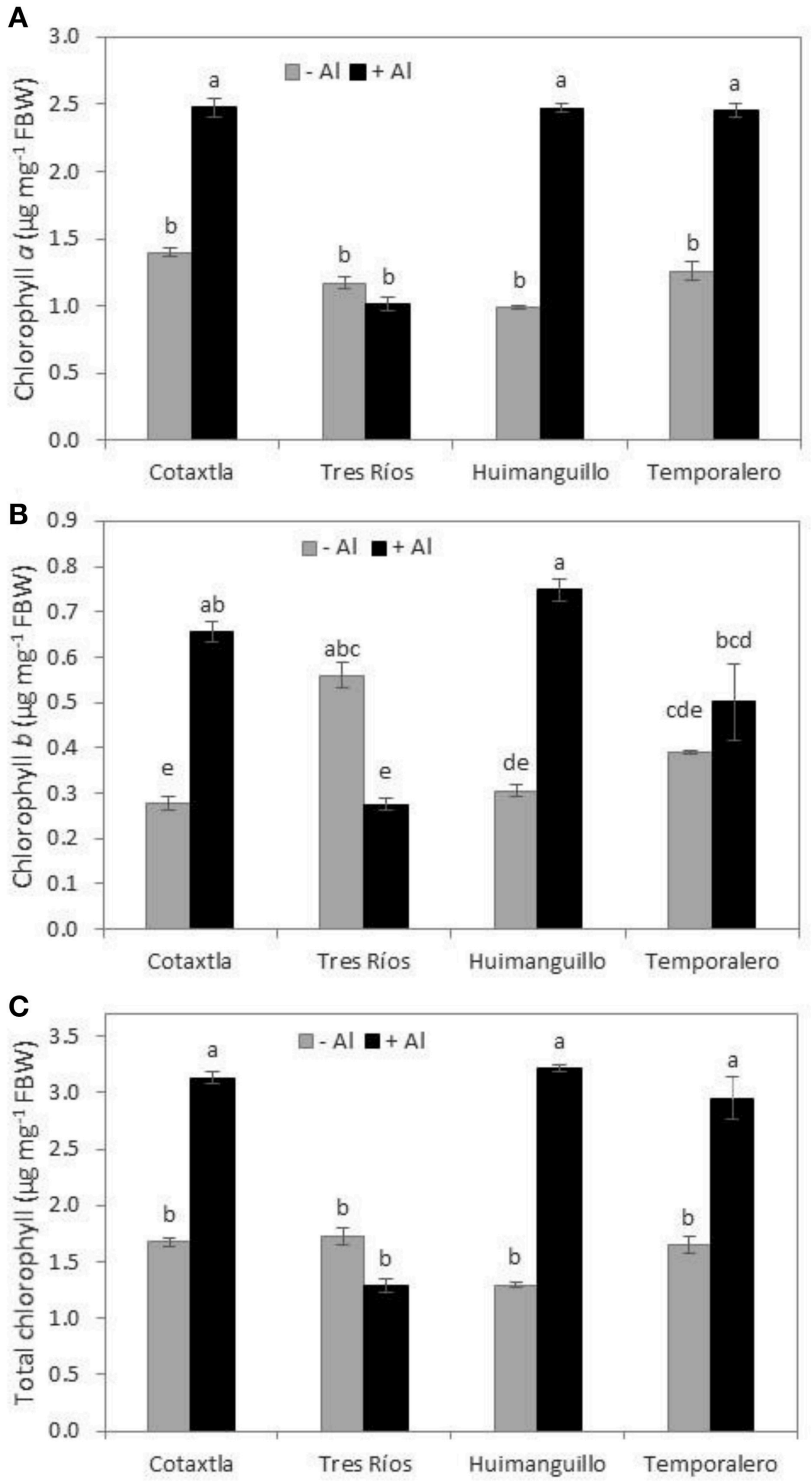
**Chlorophyll concentrations in rice plant leaves in response to Al treatment**. Chlorophyll *a* **(A)**, Chlorophyll *b* **(B)**, and total Chlorophyll **(C)** in the 2nd and 3rd youngest leaves of rice plant cultivars Cotaxtla, Tres Ríos, Huimanguillo and Temporalero grown in the absence (−) or presence of 200 μM Al (+) for 20 days. Values are means ± SE from five individual plants. Different letters above the column indicate significant differences (Tukey, *P* ≤ 0.05). FBW, fresh biomass weight.

Chlorophyll *a, b* and total chlorophyll concentrations increased as a consequence of Al treatment in all four cultivars tested, with the exception of Tres Ríos (Figure 3). In particular, in Al-treated Cotaxtla, Huimanguillo and Temporalero plants, chlorophyll *a* concentrations were 50% higher than in the control. In Tres Ríos, chlorophyll *a* concentration was not affected by Al, though in Al-treated plants it was lower than in the other cultivars (Figure 3A). Chlorophyll *b* concentration was higher in Al-treated Cotaxtla and Huimanguillo plants, in comparison to control plants; in Temporalero plants there was no Al effect, whereas in Tres Ríos plants there was a reduction in chlorophyll *b* in comparison to the control (Figure 3B). Regarding total chlorophyll concentrations, there was a similar behavior to that showed in chlorophyll *a*, with Tres Ríos being unaffected by Al, whereas the rest of the cultivars showed a significant increase in Al-treated plants in comparison to the control (Figure 3C).

### Total free amino acids and proline concentrations are not affected by Al, but soluble sugars are

**Figure 4:**
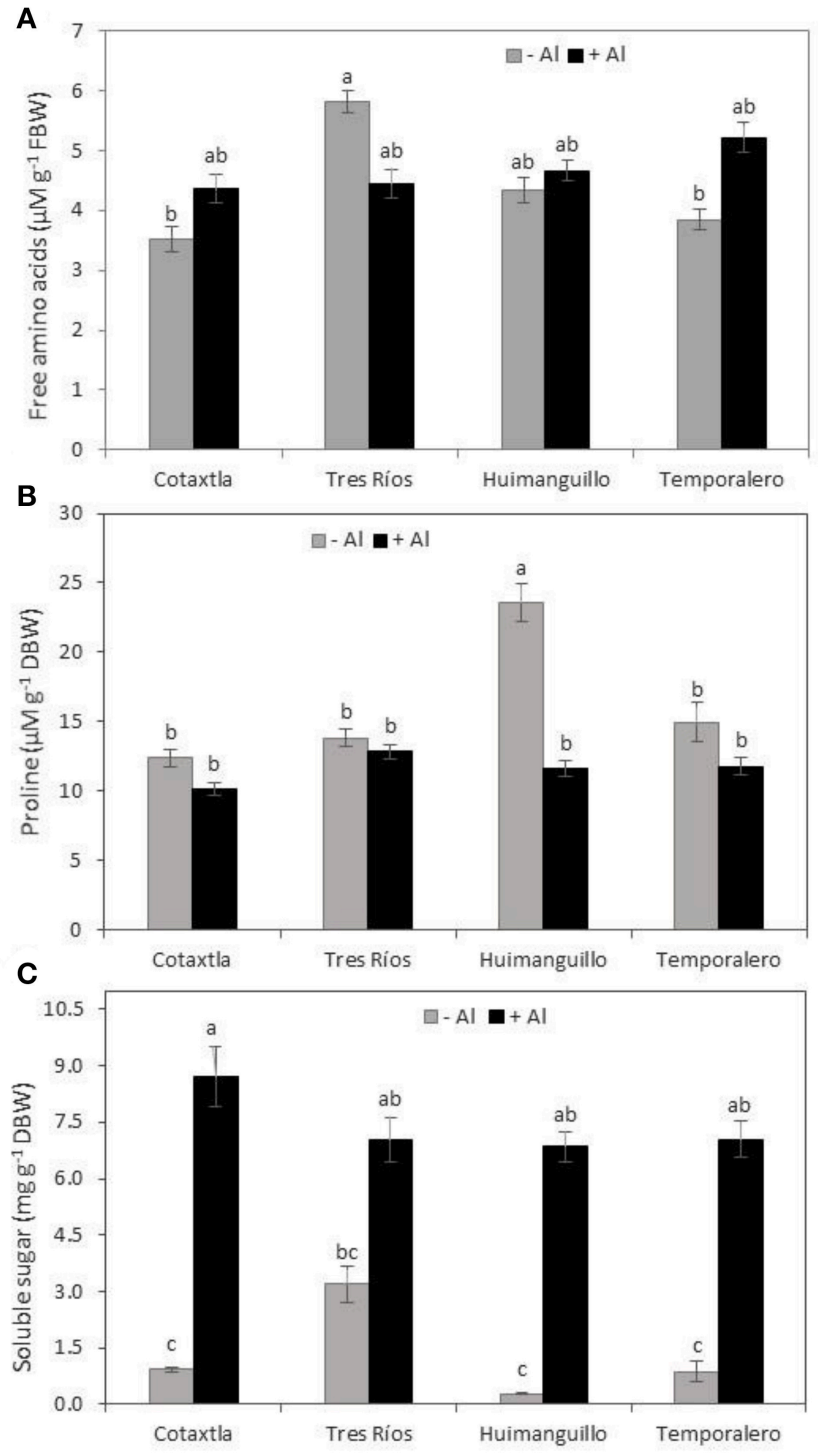
**Free amino acids, proline and soluble sugars concentrations of rice plants in response to Al treatment**. Total free amino acids **(A)** in the 2nd and 3rd youngest leaves, proline **(B)** and soluble sugar **(C)** in leaves of rice plant cultivars Cotaxtla, Tres Ríos, Huimanguillo, and Temporalero grown in the absence (−) or presence of 200 μM Al (+) for 20 days. Values are means ± SE from five individual plants. Different letters above the column indicate significant differences (Tukey, *P* ≤ 0.05). FBW, fresh biomass weight; DBW, dry biomass weight.

In order to investigate whether Al causes a stressful effect on the rice cultivars evaluated, we determined amino acids and proline concentrations in shoots. No differences were found between control and Al-treated plants concerning amino acid concentrations, in all four rice cultivars evaluated. Nonetheless, we did observe that Tres Ríos plants displayed the highest concentration of free amino acids in the control, while Cotaxtla and Temporalero had the lowest under the same environmental conditions (Figure 4A). Similarly, Al did not affect proline concentration in leaves of Cotaxtla, Tres Ríos, and Temporalero, whereas Huimanguillo plants displayed almost double the concentration of proline in the control in comparison to Al-treated plants (Figure 4B).

Surprisingly, total soluble sugars concentration was significantly increased in Al-treated plants (Figure 4C). Indeed, in Cotaxtla plants, total soluble sugars concentration was nine-fold higher than that showed by the control, whereas in Tres Río it was two-fold increased, in Huimanguillo it was 25 times higher, and in Temporalero this increase was eight-fold higher than that showed by control plants (without Al).

### Root and shoot aluminum and macronutrient concentrations are differentially affected by Al treatment

**Figure 5:**
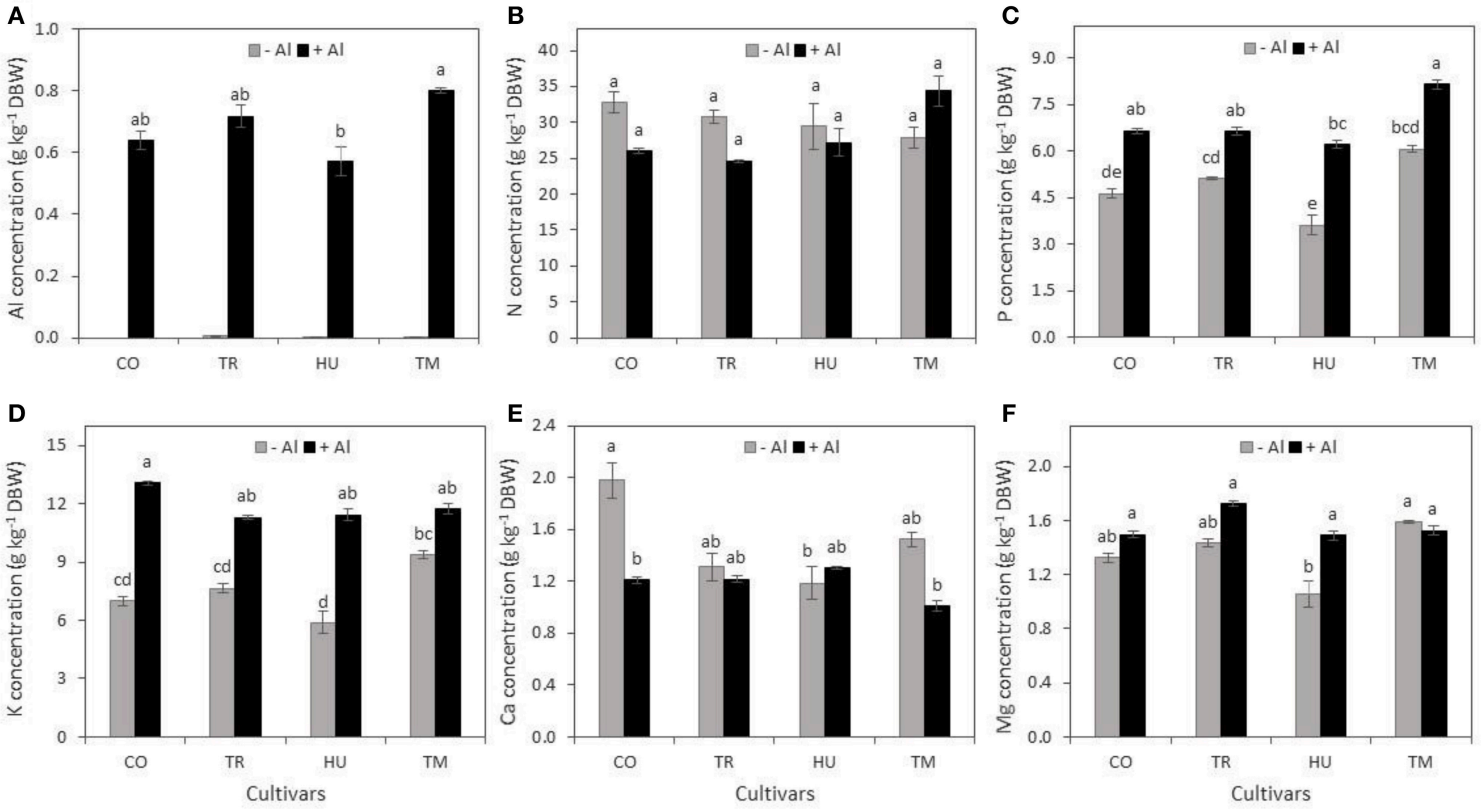
**Concentrations of aluminum and macronutrients in roots of rice plants in response to Al treatment**. Concentration of Al **(A)**, N **(B)**, P **(C)**, K **(D)**, Ca **(E)**, and Mg **(F)** in roots of rice plant cultivars Cotaxtla (CO), Tres Ríos (TR), Huimanguillo (HU), and Temporalero (TM) grown in the absence (−) or presence of 200 μM Al (+) in the nutrient solution for 20 days. Values are means ± SE from five individual plants. Different letters above the column indicate significant differences (Tukey, *P* ≤ 0.05). DBW, dry biomass weight.

Aluminum concentrations in root tissues were clearly increased in Al-treated plants; in control plants Al concentrations were nearly non-existent. When comparing cultivars, we observed that Temporalero (TM) showed a higher concentration of Al in roots, while Cotaxtla (CO) and Tres Ríos (TR) displayed a similar Al concentration in this tissue; finally, Huimanguillo (HU) registered the lowest Al concentration in roots (Figure 5A).

Regarding N concentrations we did not find any difference among cultivars, nor between Al treatments (Figure 5B). In roots all four cultivars exposed to Al, P concentrations increased, in comparison to the control (−). Among cultivars, TM plants displayed the highest concentration of P and in HU roots the lowest concentration of this macronutrient was recorded (Figure 5C). A similar trend was observed regarding K concentration in roots, since three cultivars except TM increased K concentrations (Figure 5D).

As for Ca, we only observed a reduction in roots of CO plants exposed to Al (+), in comparison to control plants (−), whereas in the rest of the cultivars evaluated we were unable to find significant effects of Al (Figure 5E).

Magnesium concentration in roots was similar in almost all cultivars except HU, where its concentration increased in response to Al treatment, in comparison to control plants (Figure 5F).

**Figure 6:**
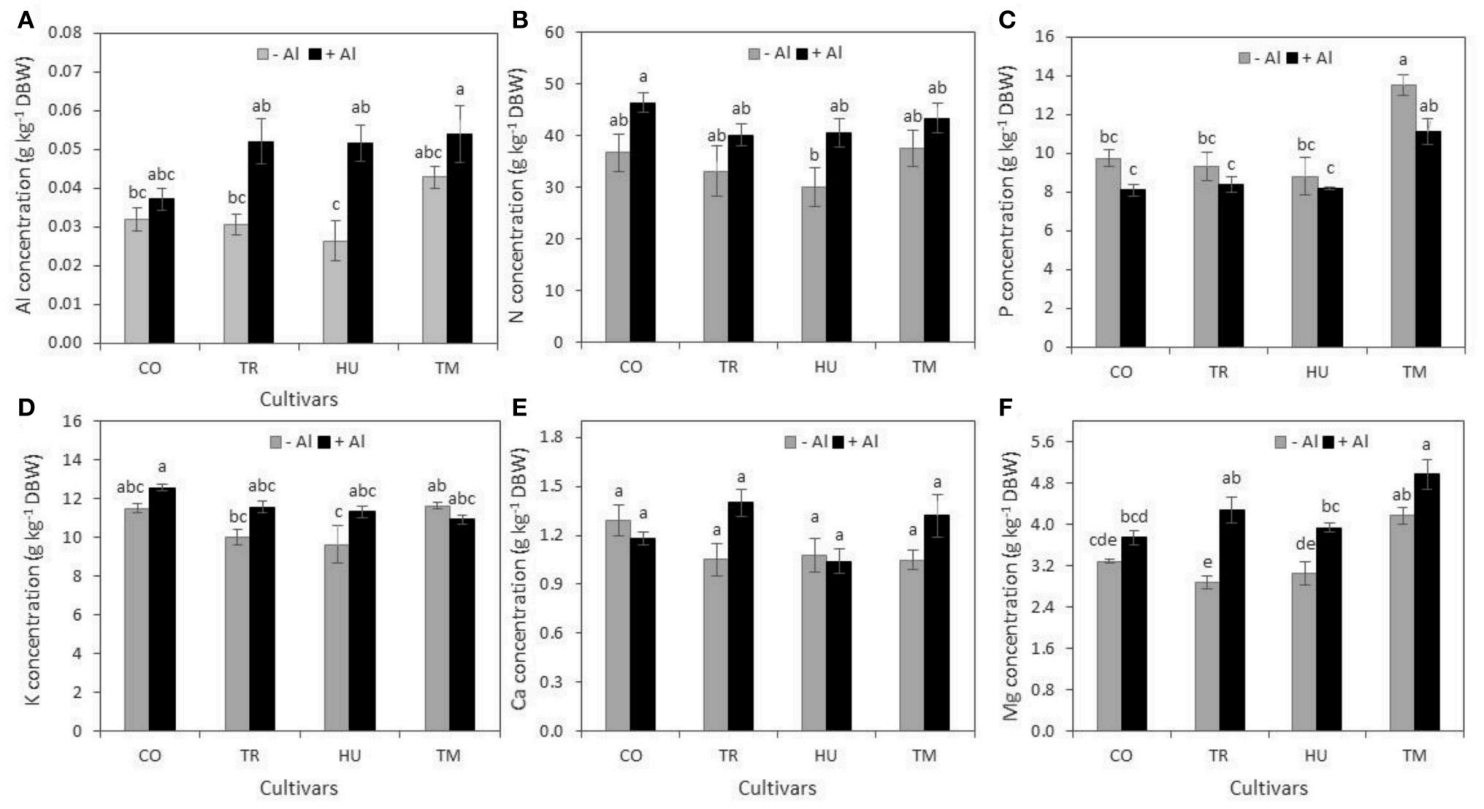
**Concentration of aluminum and macronutrients in shoots of rice plants under Al treatment**. Concentration of Al **(A)**, N **(B)**, P **(C)**, K **(D)**, Ca **(E)**, and Mg **(F)** in shoot of rice plant cultivars Cotaxtla (CO), Tres Ríos (TR), Huimanguillo (HU) and Temporalero (TM) grown in the absence (−) or presence of 200 μM Al (+) in the nutrient solution for 20 days. Values are means ± SE from five individual plants. Different letters above the column indicate significant differences (Tukey, *P* ≤ 0.05). DBW = dry biomass weight.

Aluminum and macronutrient concentrations in shoots are shown in Figure 6. As expected, Al concentration in shoots of the four cultivars evaluated increased in Al-treated plants in comparison to the control. Nevertheless, this increase was only significant for HU plants (Figure 6A).

Nitrogen concentration in shoots was not significantly affected by the treatments tested (Figure 6B). The same tendency was observed regarding P concentration, though TM plants showed the highest P concentration in both Al-treated and control plants (Figure 6C). Both K (Figure 6D) and Ca (Figure 6E) were not significantly affected by Al, and differences among cultivars were also not evident. Interestingly, in TR and HU plants there was a significant increase in Mg concentration stimulated by Al. However, TM showed the highest Mg concentrations both in Al-treated and control plants, whereas TR shoots recorded the lowest Mg concentration in the control (Figure 6F).

Nutrient solution was prepared with analytical-grade chemicals (purity ≥ 99%). Nevertheless, control plants might have received traces of Al, since we found small amounts of this element in shoots of those plants. Similar results have been reported by Marín-Garza et al. (2010), Hajiboland et al. (2013a), Gómez-Merino et al. (2014), and Roselló et al. (2015). Hajiboland et al. (2013b) attributed these responses to a possible content of Al in seeds and in the chemicals used to prepare the nutrient solution for control plants. Roselló et al. (2015) attributed the higher Al concentrations of Al in control plants to an efficient mechanism of Al exclusion in some genotypes, but not in all.

### Al induces transcription factors gene expression in roots of rice plants

We analyzed the expression pattern of 57 *NAC* genes, of which 25 showed changes in gene expression after 24 h of exposure to 200 μM Al, which represents 44% of all *NAC* genes tested. The fold change in the expression of those genes was evident in most cultivars tested (Table 1). A gene was considered Al-regulated when the log_2_ of 2^ΔΔCt^ was ≥ 2 as an absolute value. Thus, in Cotaxtla plant roots 21 genes were found induced, 19 in Tres Ríos, 18 in Huimanguillo and 24 in Temporalero. We could also observe that three genes were exclusively induced in Temporalero: *Os01g15640, Os10g21560*, and *Os04g40130*; while *Os06g51070* was only induced in Cotaxtla, Temporalero and Huimanguillo, but not in Tres Ríos. Similarly, the genes *Os03g21060, Os09g33490*, and *OsNAC5* were induced in three of the four cultivars evaluated, but they were not found to be differentially expressed in Huimanguillo plants. Of the 25 genes found to be differentially expressed upon Al exposure, 14 (54%) belong to the NAM (no apical meristem) subfamily (Table 1).

**Table 1:** **Expression levels of *NAC* genes in roots of rice plants in response to Al treatment**.

**Locus Identifier**	**Gene name**	**Relative expression (Fold change)**			
**TIGR v5.0**		**Cotaxtla**	**Tres Ríos**	**Huimanguillo**	**Temporalero**
Os02g56600	No apical meristem (NAM) protein, putative	4.52 ± 0.29a	4.40 ± 0.37a	4.84 ± 0.59a	4.39 ± 0.33a
Os03g21060	No apical meristem (NAM) protein, putative	2.39 ± 0.23b	2.50 ± 0.24b	1.40 ± 0.16c	5.17 ± 0.09a
missing annotation:	No apical meristem (NAM) protein, putative	4.66 ± 0.47a	3.78 ± 0.73ab	2.69 ± 0.51b	5.04 ± 0.31a
Os03g60080	Putative NAC-domain protein	8.59 ± 0.49a	2.37 ± 0.24b	3.12 ± 0.29b	2.16 ± 0.24b
Os10g42130	putative NAM (no apical meristem) protein	5.45 ± 0.66a	4.18 ± 0.40ab	3.38 ± 0.23b	4.06 ± 0.73ab
Os01g66490	No apical meristem (NAM) protein, putative	3.41 ± 0.68ab	2.11 ± 0.33b	3.13 ± 0.2ab	4.21 ± 0.28a
Os01g15640	No apical meristem (NAM) protein, putative	1.63 ± 0.28b	−0.32 ± 0.26c	1.34 ± 0.14b	3.26 ± 0.50a
Os07g04560	hypothetical protein	6.11 ± 0.22a	−0.38 ± 0.16d	1.64 ± 0.16c	4.25 ± 0.96b
Os09g32040	Similar to NAM like protein 7	1.53 ± 0.24b	−1.29 ± 0.31c	3.05 ± 0.54b	4.89 ± 0.70a
Os12g43530	No apical meristem (NAM) protein, putative	7.62 ± 0.65a	6.19 ± 0.88ab	3.71 ± 0.49c	4.6 ± 0.23bc
Os06g51070	NAM (no apical meristem)-like protein [imported]—*Arabidopsis thaliana*	5.43 ± 0.75a	1.51 ± 0.30b	4.26 ± 0.37a	2.60 ± 0.10b
Os09g33490	Similar to NAC domain protein NAC2	2.53 ± 0.25ab	3.65 ± 0.81a	1.89 ± 0.44b	3.30 ± 0.30ab
Os11g31330	No apical meristem (NAM) protein, putative	6.30 ± 0.71a	6.17 ± 0.38a	2.51 ± 0.23c	4.38 ± 0.38b
Os04g35660	No apical meristem (NAM) protein, putative	3.82 ± 0.40ab	3.59 ± 0.41b	3.21 ± 0.15b	4.86 ± 0.40a
missing annotation:	No apical meristem (NAM) protein, putative	5.25 ± 0.35a	4.26 ± 0.17ab	3.43 ± 0.45b	1.68 ± 0.15c
Os03g59730	Putative No apical meristem (NAM) protein	5.35 ± 0.13a	4.43 ± 0.54ab	3.82 ± 0.01b	3.72 ± 0.23b
Os07g13920	No apical meristem (NAM) protein, putative	4.87 ± 0.34b	5.90 ± 0.20a	2.63 ± 0.23d	3.82 ± 0.33c
Os10g21560	putative transcription factor	1.21 ± 0.26b	−0.77 ± 0.17c	1.48 ± 0.12b	4.61 ± 0.14a
Os04g40130	Similar to probable salt-inducible protein [imported] - *Arabidopsis thaliana*	1.56 ± 0.12b	1.04 ± 0.40b	1.72 ± 0.22b	3.56 ± 0.35a
Os08g10080	Similar to NAC domain protein NAC1	5.10 ± 0.23a	4.50 ± 0.72a	3.91 ± 0.56a	4.18 ± 0.69a
Os12g29330	Similar to NAC domain protein NAC2	4.70 ± 0.66ab	4.74 ± 0.43ab	3.08 ± 0.48b	5.35 ± 0.56a
Os04g38720	OsNAC2 protein	4.16 ± 0.36a	4.78 ± 0.54a	4.66 ± 0.32a	4.20 ± 0.53a
Os11g08210	OsNAC5 protein [imported] - rice	4.76 ± 0.55ab	4.83 ± 0.06a	3.15 ± 0.53b	5.13 ± 0.66a
Os11g08210	OsNAC5	3.53 ± 0.70b	2.95 ± 0.18b	1.31 ± 0.24c	5.31 ± 0.52a
Os01g66120	OsNAC6	4.96 ± 0.52a	3.43 ± 0.50ab	4.77 ± 0.40a	2.86 ± 0.55b

*Twenty-four-day-old Cotaxtla, Tres Ríos, Huimanguillo and Temporalero rice plants were subjected to Al treatment (200 μM Al), for 24 h. Gene expression was quantified using log_2_ from method 2^−ΔΔCt^, and Actin (Os03g50890) was used as a reference gene for data normalization. Values are means ± SE from three independent biological replicates. Different letters in each row indicate significant differences (Fisher LSD test; P ≤ 0.05)*.

**Figure 7:**
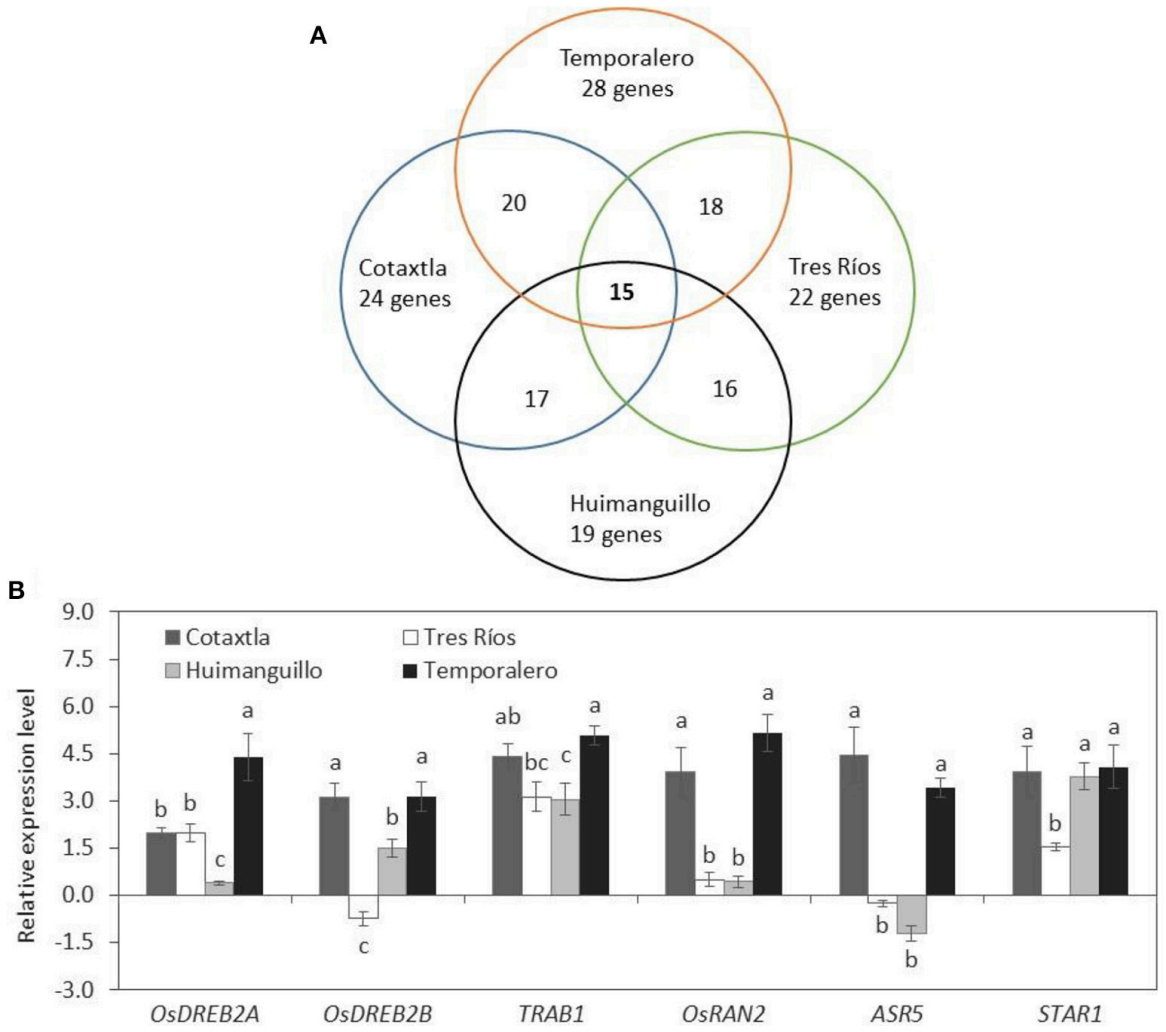
**Expression analysis of Aluminum-regulated genes in rice**. Venn diagram depicts the number of Al-responsive *NAC* genes in roots of four rice cultivars **(A)**. Relative expression level of other transcription factors and previously reported Al-responsive genes in Al-treated rice plants are shown **(B)**. Total RNA was extracted from roots of rice plant cultivars Cotaxtla, Tres Ríos, Huimanguillo, and Temporalero grown in the absence (−) or presence of 200 μM Al (+) in the nutrient solution for 24 h. Relative gene expression was quantified using the log_2_ from method 2^−ΔΔCt^, and *Actin* (Os03g50890) was used as a reference gene for data normalization. The values are mean ± SE from three independent biological replicates. Different letters above the column indicate significant differences between cultivars evaluated (Fisher LSD test; *P* ≤ 0.05).

Furthermore, 20 genes were regulated both in Cotaxtla and Temporalero, 17 in Cotaxtla and Huimanguillo, 16 in Huimanguillo and Tres Ríos, and 18 in Tres Ríos and Temporalero. Of the 57 *NAC* genes evaluated, 15 genes were induced in all four cultivars tested, representing 60% of the total number of genes induced by Al in our study (Figure 7A). Moreover, we found that the gene *OsDREB2A* was only induced in Temporalero roots, whereas *OsDREB2B1* and *TRAB1* were upregulated in the four cultivars, though with a higher level in Cotaxtla and Temporalero. *OsRAN2* was induced in Cotaxtla and Temporalero, but not in Tres Ríos or Huimanguillo (Figure 7B). In plants treated with Al, our positive control *ASR5* was induced in roots of Cotaxtla and Temporalero, while slightly repressed in Tres Ríos and Huimanguillo. Instead, the expression of *STAR1* was induced in all four cultivars evaluated, but its expression was stronger in Cotaxtla, Huimanguillo and Temporalero.

## Discussion

### Aluminum improves growth and stimulates soluble sugars concentrations

Plant species differ in their response to Al exposure, and rice has largely been found to be one of the most tolerant crops to toxic levels of Al (Famoso et al., 2010). In this study we confirmed that rice is indeed highly tolerant to Al, and proved that our four cultivars increased plant height (Figure 1A), root length (Figure 1D), and biomass production (Figure 2) upon exposure to 200 μM Al. In a previous study, Marín-Garza et al. (2010) evaluated growth parameters in the cultivars Tres Ríos, Huimanguillo and Temporalero in response to 0, 200, and 400 μM Al, though they found no significant differences regarding plant height. Subsequently, Gómez-Merino et al. (2014) reported that plant growth was significantly affected by 400 μM Al, whereas with 200 μM Al root length was similar to the control (no Al added). Interestingly, Temporalero plants increased root length with 200 μM Al, though this increase was not significant when compared to the control (Gómez-Merino et al., 2014). The differences found in our study in comparison to the results reported by Marín-Garza et al. (2010) and Gómez-Merino et al. (2014) may be attributed to the general management of the experiments: seed germination conditions (MS medium vs. filter paper in Petri dishes irrigated with distilled water); the nutrient solution employed for the hydroponic assay (Yoshida vs. Steiner); acclimation period in the nutrient solution before Al treatment application (2 weeks vs. 1 week); and age of plants before exposure to Al (24 days vs. 35 days). Similar results on the beneficial effect of Al in promoting growth in other plant species have been reported. For instance, tea plants (*Camellia sinensis*) exposed to 100 μM Al developed better than those grown in Al-free media (Hajiboland et al., 2013a). In rice cultivar OM4900, Nhan and Hai (2013) found an increase of 35% in shoot height in plants exposed to 400 μM Al for 8 days. Moreover, Famoso et al. (2011) reported that exposure to 160 μM Al triggered significant root elongation in rice, which is similar to our results. This may be due to the fact that Al diminishes H^+^toxicity when pH is low, as a consequence of an electrostatic shift in the cell membrane surface (Poschenrieder et al., 2015).

The stimulant effect of Al has also been reported in tea plants, where this element enhances biomass production both in shoots and roots, which is similar to our results (Figure 2). Even with a higher Al concentration (i.e., 300 μM Al) than that tested here, Al-treated plants produced three-fold more biomass than control plants (Hajiboland et al., 2013a,b). A similar response was observed in tea plant suspension cells exposed to 0, 50, and 500 μM Al (Ghanati et al., 2005). In tea plants, Hajiboland et al. (2013b) also reported that the application of 300 μM Al boosts chlorophyll biosynthesis in young leaves, but not in old leaves. Likewise, our experiments also demonstrated that chlorophyll concentrations increased in young leaves of Al-treated Cotaxtla, Huimanguillo and Temporalero plants (Figures 3A–C, respectively). These results are also in full agreement with those reported by Nhan and Hai (2013) in rice plant cultivar OM4900, since both chlorophyll *a* and *b* were significantly higher in plants treated with 200, 300, 400, and 500 μM AlCl_3_, as compared with the control.

When exposed to stress conditions, plants tend to accumulate free amino acids, especially proline (Hayat et al., 2012). We found that 200 μM Al does not represent a stressful factor for the rice cultivars tested. Instead, we observed a stimulant effect on most variables measured, and would expect that both amino acids and proline were similar in Al-treated and control plants. We confirmed this hypothesis, since none of the cultivars assayed displayed significant differences regarding either amino acids (Figure 4A) or concerning proline (Figure 4B) concentrations in shoots. Similarly, in tea plants Al stimulated growth and biomass production, whereas amino acid concentrations were not affected (Hajiboland et al., 2013b). Since amino acids are precursors of proteins, no changes in the concentrations of amino acids in response to Al may indicate that there was no degradation of proteins in Al-treated plants. Hajiboland et al. (2013b) also report a rise in proline concentration in leaves and roots of tea plants treated with 300 μM Al, which was attributed to the important role of proline in removing free radicals in response to the acidic media triggering oxidative stress. Under our experimental conditions, we did not find different responses in proline concentrations in Al-treated and control plants, with the exception of Huimanguillo, which showed a reduction in proline concentration in response to Al. This behavior could mean that this cultivar, in particular, possesses a less efficient antioxidant mechanism in comparison to the rest of the cultivars tested. Importantly, it has been reported that proline concentration is not always correlated with stress tolerance (Szabados and Savouré, 2010).

Likewise, total soluble sugars concentration may be closely correlated with tolerance to saline stress (Kong et al., 2011; Zhang et al., 2015). Herein we found that Al significantly increased soluble sugars in rice shoots. These results are different from those reported in tea plants, since young leaves and roots showed similar concentrations of such carbohydrates both in Al-treated and control plants, while in old leaves soluble sugars were reduced in response to Al exposure (Hajiboland et al., 2013b). Similar results to those reported herein have been observed in sunflower varieties Sirena and Sanbero, since exposure to 100 and 200 μM Al significantly increased soluble sugars concentration (Ziaei et al., 2014). Soluble sugars do not only function as metabolic resources and structural constituents of cells, but also act as signals regulating various processes associated with plant growth and development (Rosa et al., 2009). Hence, Al increases soluble sugars concentration in plants, which in turn may enhance growth and biomass production in rice under our experimental conditions.

### Aluminum alters macronutrients concentration in rice plants

All four rice cultivars evaluated displayed similar Al concentrations in roots (0.68 g kg^−1^ DBW on average), with Huimanguillo showing the lowest and Temporalero the highest concentrations and differences between Al-treated and control plants were significant (Figure 5A). Nonetheless, in shoots, Al concentrations showed less differences between Al-treated and control plants (Figure 6A) and such values were indeed much lower than those found in roots (Figure 5A). In fact, Al concentrations in shoots ranged from 0.026 g kg^−1^ DBW in Huimanguillo plants under control conditions (the lowest value in shoots), to 0.054 g kg^−1^ DBW in Temporalero plants treated with Al (the highest value found in shoots). Interestingly, Cotaxtla and Temporalero displayed statistically similar values of Al concentrations in shoots in Al-treated and control plants, whereas Huimanguillo and Tres Ríos showed almost double the concentration of Al in Al-treated plants, in comparison to the control. This response suggests that the first two cultivars (Cotaxtla and Temporalero) have developed more efficient mechanisms to restrict Al transport to the shoots, in comparison to the last two cultivars (Huimanguillo and Tres Ríos). Likewise, Roselló et al. (2015) reported that comparison of root and shoot Al concentrations between Nipponbare (Al-tolerant) and Modan (Al-sensitive) varieties demonstrated that the basis of the Al resistance strategy in Nipponbare is the avoidance of Al uptake into the roots and an efficient restriction of Al transport to the shoots. Modan was able to restrict Al translocation to the shoots only during the first 24 h of Al exposure. Then Al shoot concentrations increased reaching 2.5 times higher concentrations than the corresponding background values in the control plants. Contrastingly, Al concentrations were not enhanced in the shoots of Nipponbare during the 72 h exposure time. Poschenrieder et al. (2015) argue that contrasting responses among species and varieties can be explained by three different mechanisms: (1) the amelioration of H^+^ toxicity by Al^3+^; (2) preventing Al to reach the target sites and damage; and (3) a putative (still unknown) mechanism that apparently implies a restructuring of the cell wall in the root tip after an initial highly sensitive response (activation of defense genes).

Aluminum exposure for long periods may lead to nutrient limitations, among which Ca, Mg, N (in the form of NH_4_), P and K are the most common deficiencies in acid soils with toxic levels of Al (Lenoble et al., 1996; Mariano and Keltjens, 2005). Interestingly, under our experimental conditions we did not find any deficiency in relation to the macronutrients N, P, K, Ca, and Mg, neither in roots (Figure 5) nor in shoots (Figure 6). Similar results have been reported by Marín-Garza et al. (2010), since they did not find any deficiency of Ca, K, Mg, and P in roots of cultivars Temporalero, Huimanguillo, and Tres Ríos grown either in 0 or 200 μM Al containing solutions, though in Tres Ríos there was a reduction in Ca concentration. One of the nutrients most affected by Al is P, since it forms an Al-P complex of very low solubility, which reduces P-availability in acid soils with high levels of toxic Al (Haynes and Mokolobate, 2001). Even in acid soils with high concentrations of P, the availability of this nutrient is highly restricted (Fukuda et al., 2007). Surprisingly, herein we found a synergic effect of Al on P, since P concentrations in roots of plants grown with 200 μM Al were higher than those found in control plants (Figure 5C); in shoots we were unable to find significant effects of Al on P concentrations (Figure 6C). A similar response was observed regarding K concentrations (Figures 5D, 6D). However, studies on the relationships between Al and K have produced controversial results. For instance, while Matsumoto and Yamaya (1986) and Nichol et al. (1993) observed that Al inhibits K uptake, Lindberg (1990) and Tanoi et al. (2005) reported an enhanced uptake of K driven by Al. This response could be attributed to a reduction in the efflux of K, instead of increased absorption (Sasaki et al., 1995). On the other hand, it has been observed that toxic effects of Al cause Ca deficiencies (Rengel and Elliott, 1992). Furthermore, Al affects Ca cell homeostasis in plants (Bose et al., 2015). Nevertheless, it has also been reported that Al inhibits root hair growth without affecting Ca influx in *Limnobium stoloniferum* (Jones et al., 1995). These findings, at least in part, coincide with our results, since with the exception of Cotaxtla we did not observe differences in Ca concentrations either in roots or shoot of plants grown in absence or presence (200 μM Al) of the metal (Figures 5E, 6E). Conversely, in Cotaxtla plants we observed a reduction of Ca concentration in roots in response to Al treatment (Figure 5E), though root growth was also enhanced (Figure 1D), as well as root biomass production (Figures 2C,D). It has also been reported that Al inhibits Mg uptake in *Lolium multiflorum* exposed to 26 μM Al. This inhibition may be driven by a competitive interaction between Al and Mg for Mg transporters located in the plasma membrane (Rengel, 1990). On the contrary, our results demonstrate that Al-treated plants displayed a Mg concentration similar or even higher in Al-treated plants in comparison to control plants, both in roots (Figure 5F) and shoots (Figure 6F). Summarizing, Al treatment did not affect N and Ca concentrations in roots and shoots, or P and K in shoots; it had a synergic effect with P and K in roots, as well as with Mg in some cultivars (in roots of Huimanguillo and in shoots of Tres Ríos and Huimanguillo). Accordingly, Bose et al. (2015) argue that plants with the capacity of increasing P, Ca and Mg uptake show better growth under Al-stress conditions since they can absorb higher amounts of H^+^ and at the same time prevent cytoplasmic acidification. Moreover, we confirm that Al as a beneficial element can increase availability of P, which in turn is absorbed by roots in higher amounts.

### Expression level of *NAC* transcription factor genes is regulated by Al

Transcription factors are proteins that bind to specific DNA sequences, thereby controlling the rate of transcription of genetic information from DNA to messenger RNA. They carry out their functions alone or with other proteins in a complex, by promoting (as an activator) or blocking (as a repressor) the recruitment of RNA polymerase to specific genes. Therefore, transcription factors play a pivotal role in modulating plant responses to environmental stimuli and stress agents, since they lead signaling cascades aimed at boosting expression of target genes, including those involved in Al metabolism and tolerance mechanisms (García-Morales et al., 2013; Garcia-Oliveira et al., 2015). The main transcription factors involved with Al tolerance in plants characterized so far belong to the C2H2-type zinc-finger family, which contain WRKY and Abscisic Acid, Stress and Ripening (ASR) domains (Arenhart et al., 2014; Yokosho and Ma, 2015). Members of this family such as WRKY46 and ASR5 are also involved in tolerance mechanisms against other stress factors (Yokosho and Ma, 2015). Concerning NAC transcription factors, they are widespread in the plant kingdom (i.e., 117 in Arabidopsis and 151 in rice) (http://plntfdb.bio.uni-potsdam.de/v3.0/) and have been reported to be involved in vital processes in plant cells, including growth, development and responses to environmental stimuli and stressors. Indeed, a large amount of *NAC* genes have been implicated in both biotic and abiotic stress responses. For instance, the genes *OsNAC5, OsNAC6, OsNAP*, and *SNAC1* in rice are induced by drought, salinity and cold stress (Nakashima et al., 2007; Takasaki et al., 2010; Saad et al., 2013; Chen et al., 2014). Herein we have demonstrated that two of those genes, *OsNAC5* and *OsNAC6*, were also induced by Al treatment (Table 1). Importantly, an EST (Accession CA095885) similar to *OsNAC5* was detected in an expression profiling analysis aimed at identifying Al-regulated genes in maize (Cançado et al., 2008), which further supports our findings. In our analysis, the expression *OsNAC5* was found to be induced in response to Al in all four rice cultivars evaluated, though the highest level of induction was observed in Temporalero and the lowest in Huimanguillo. Our positive controls *ASR5* and *STAR1* also responded to Al under our experimental conditions.

Furthermore, we report that 14 *NAC* genes previously reported to be regulated by NaCl (100 mM) (García-Morales et al., 2014) are also regulated by Al. Moreover, the gene *Os04g38720* was induced by Al in the four cultivars evaluated, and has been reported to be induced by cold too (Yun et al., 2010). The gene *Os03g21060*, which was previously reported to be induced by cold in Nipponbare (Yun et al., 2010), was also found to be induced by Al in the cultivars Cotaxtla, Tres Ríos and Temporalero. Just recently, Wang et al. (2016) reported that expression of the tomato *NAC* transcription factor *SlNAC35* was induced by drought stress, salt stress, bacterial pathogens and signaling molecules, suggesting its involvement in plant responses to biotic and abiotic stimuli. Therefore, NAC transcription factors mediate in a wide range of biological processes, including growth, development and responses to environmental cues of both a biotic and abiotic nature. Herein we report for the first time that *NAC* genes are induced by Al in rice, which open new avenues in the signal transduction pathways connecting this metal and the transcriptional machinery triggered by NAC proteins. Nevertheless, the molecular machinery underlying these pathways remains to be elucidated. In order to gain insight into these gaps, we are currently performing a comprehensive analysis of *NAC* genes promoters and their underlying mechanisms in transcriptional regulation, including epigenetic marks and chromatin-based regulation.

In addition to the *NAC* transcription factor genes tested, we also measured the expression pattern of other transcription factors belonging to the dehydration responsive element binding (DREB) family. *OsDREB2A* and *OsDREB2B1* genes are responsive to low temperature, drought and salinity (Matsukura et al., 2010). The former showed Al-inducible gene expression in Temporalero plants, whereas the latter was induced in Cotaxtla and Tres Ríos plants in response to Al (Figure 7B). Interestingly, the overexpression of *OsDREB2A* in soybean (*Glycine max*) induced the expression of key genes involved in abiotic stress responses and drove significant increases in soluble sugars and proline concentrations, which in turn improved plant performance upon salt stress (Zhang et al., 2013). The gene *TRAB1* is a member of the Basic Leucine Zipper (bZIP) proteins involved in abscisic acid signaling and stress responses (Yang et al., 2011). We found this gene to be induced by Al, mainly in Temporalero and at a lower level in Huimanguillo (Figure 7B). Finally, *OsRAN2* is a small GTPase, which when overexpressed in rice and Arabidopsis renders transgenic plants hypersensitive to salinity and osmotic stress (Zang et al., 2010) as well as cold stress (Xu and Cai, 2014). This gene was induced in Cotaxtla and Temporalero exposed to Al, suggesting a possible role of this GTPase in Al metabolism and signaling. In order to further validate our results, we also analyzed the expression of two genes previously reported as Al-responsive: *ASR5 and STAR1*. The gene *ASR5* (*Abscisic acid, stress, and ripening*) is expressed in chloroplasts, cytoplasm and nucleus, and rice plants with silenced *ASR* genes are highly sensitive to Al (Arenhart et al., 2013, 2014, 2016). In our study, the expression of *ASR5* in Al-treated plants was induced in Cotaxtla and Temporalero, but not in Tres Ríos and Huimanguillo (Figure 7B). Similarly, in the Al-sensitive cultivar Taim, *ASR5* was not differentially regulated in plants exposed to Al (Freitas et al., 2006). Instead, Roselló et al. (2015) reported a slight induction of this gene in rice plants exposed to 500 μM Al, but no differences were observed between subspecies (japonica cv. Nipponbare and indica cv. Modan). Importantly, ASR5 may act as a transcriptional regulator of multiple Al-responsive genes in rice, including *STAR1* (Arenhart et al., 2014, 2016). *STAR1* (*sensitive to aluminum rhizotoxicity 1*) encodes a nucleotide binding domain of a bacterial-type ATP binding cassette (ABC) transporter, and is mainly expressed in roots of both Kishihikari wild-type and the mutant *star1* rice plants (Huang et al., 2009). Under our experimental conditions, *STAR1* was induced in three of the four rice cultivars evaluated (i.e., Cotaxtla, Huimanguillo and Temporalero) 24 h after exposure to 200 μM Al (Figure 7B). Roselló et al. (2015) found that *STAR1* induction increased according to time period (from 0 to 48 h of exposure to Al) in Nipponbare (japonica) plants, whereas in Modan (indica) the expression of this gene reached a maximum 24 h after Al exposure, and subsequently its expression decreased to values similar to those of the control (no Al added).

Transcription factors here evaluated have been shown to be differentially regulated by Al. Although deducing the biological role of their encoded proteins in Al metabolism and signaling remains a daunting challenge, herein we report for the first time that such genes are transcriptionally activated by a beneficial element inducing hormesis in rice. Interestingly, *NAC* genes have been identified in the genomes of important crop species such as grape, soybean, Chinese cabbage, maize, apple, potato, banana, tobacco, tomato and cassava (Shao et al., 2015). Nonetheless, further research is still needed to determine their particular physiological functions and to evaluate their potential as biotechnological tools to improve and expand the use of beneficial elements like Al. Importantly, beneficial elements have been postulated as key components for improving crop plant productivity and yield quality in light of global challenges such as climate change and increasing food demand. As a consequence of climate change, the impact of environmental stressors of both a biotic and abiotic nature hinders plant growth and agricultural productivity (Moyer, 2010). In fact, climate prediction models forecast that crop plants will have to cope with more stress factors occurring simultaneously in the future. Since NAC transcription factors have been shown to be commonly induced by multiple stresses, they represent promising candidates to breed broad-spectrum stress tolerant crops in order to meet increasing demand for food productivity under adverse agricultural conditions (Shao et al., 2015).

In conclusion, Al can provide outright stimulation to rice that might not occur with other crops. However, further research is required to find the right method of application (i.e., hydroponic solution, foliar spray, nanofertilizers, etc.), source, rate and phenological stage of Al application for different rice genotypes.

Because of environmental concerns and the narrow range between the stimulating and toxic concentrations of Al, its practical use merits further research. However, studies of Al as a biostimulant have contributed to the increasing awareness of the relevance of this beneficial element for the efficient activation of plant growth. The connection between Al as a beneficial element in rice and the role of NAC transcription factors as key activators of Al signaling and integrating multiple stress responses will be essential for the development of broad-spectrum stress tolerant crop plants in the near future.

## Author contributions

FG, SG, and LT developed and designed the experiments. FG and LT supervised the experiments. SG and MM carried out the physiological, biochemical and molecular analyses. LT was responsible for the nutrient analyses in plant tissues. SG, JH, and FG performed the data analyses. SG and FG wrote the manuscript. FG and LT revised the manuscript. MM and SG contributed equally to this work.

### Conflict of interest statement

The authors declare that the research was conducted in the absence of any commercial or financial relationships that could be construed as a potential conflict of interest.
