# Supplementary material for: Aluminum Enhances Growth and Sugar Concentration, Alters Macronutrient Status and Regulates the Expression of NAC Transcription Factors in Rice
Source: Front Plant Sci. 2017 Feb 14;8:73. doi: 10.3389/fpls.2017.00073 (PMC5306397; doi:10.3389/fpls.2017.00073)
Supplement: Supplementary file 1 [file Table1.doc]

# Aluminum enhances growth and sugar concentration, alters macronutrient status and regulates the expression of *NAC* transcription factors in rice

Marcos Moreno-Alvarado^1^, Soledad García-Morales^1,3^, Libia Iris Trejo-Téllez^2^, Juan Valente Hidalgo-Contreras^1^, Fernando Carlos Gómez-Merino^1^*

SUPPLEMENTARY MATERIAL

**Supplementary Material S1**. Specific primers used for the qRT-PCR analysis of rice gene expression.

| **Locus Identifier TIGR v5.0** | **Forward Primer (5'-3')** | **Reverse Primer (5'-3')** | **Reference** |
| --- | --- | --- | --- |
|
| *Os03g56580* | AGACCGACTGGATGATGCACGA | TGCAGATGGTCCAGACCTCAGCTT | Caldana et al. 2007 |
| *Os06g46270* | TGAAGTTGCCTCTCAAGGAGGACT | CAGCTTGGCAATGGTTGTCCTACT | Caldana et al. 2007 |
| *Os02g56600* | TACCAGATGTGGCGAAGCTG | TACTTGCGGTCGCGGAA | Caldana et al. 2007 |
| *Os02g06950* | CTGGCAGCTAGACTACATGCAGG | GCCATTGATGTAACGAGGAGGA | Caldana et al. 2007 |
| *Os02g42970* | GAAAACGGCACCACACCTGA | GCCACCCGCTTCTTGAACA | Caldana et al. 2007 |
| *Os03g03540* | GCGACATCGATCTCTACCGC | TACCCGATCCCACAATGTTCTT | Caldana et al. 2007 |
| *Os03g02800* | CTTCCATCCAAGGTGGATGTTG | GCCAATCTTCGTGTCAAGGCT | Caldana et al. 2007 |
| *Os07g37920* | TTGCCATCGGAGTCAGCAG | TGGAGAGTTGGAACGGATGC | Caldana et al. 2007 |
| *Os03g21060* | AACGGTTCATGCGTTGGTG | CATCCCCATGTTAGAGTGGAGC | Caldana et al. 2007 |
| *Os08g33910* | GGATCTCCCAAAGCTTGCG | GAACCCAGCTGTTGTCACCC | Caldana et al. 2007 |
| *Os03g01870* | GACCTACCAGAGGTGGCGAA | TTGCTCGCTCTGTTCCTGC | Caldana et al. 2007 |
| *No anotated_1* | GGAAGAGAAATCCTTCCTGCCT | TGCTCTGGTTGCACGGTTT | Caldana et al. 2007 |
| *Os06g01480* | TGGATTTCGTTTCCACCCG | TCTTGCGGCCACCTTCTTC | Caldana et al. 2007 |
| *Os03g60080* | CATCCCCAGGAACAACAGCA | GTACATGCCCTGGATATCGTCG | Caldana et al. 2007 |
| *Os10g42130* | GCGTGATCAAGAGGCACGA | TGCTGATCATTCCTTTCGCC | Caldana et al. 2007 |
| *Os05g34310* | CCCATAATCGCAGATGTCGAA | CCCCAAATAGAGCCATGGATG | Caldana et al. 2007 |
| *Os01g66490* | GCCGGCAGCCACAACTATAG | TTGGTTGATGCGACGACG | Caldana et al. 2007 |
| *Os01g15640* | TGCAAAGTGCGATGTTCCAA | ATGGCTCGATCCTTTCCAGTG | Caldana et al. 2007 |
| *Os07g04560* | GCAACGTGAACGGTGGCTAC | GCTGCTACTGTTCCATTGCTCC | Caldana et al. 2007 |
| *Os09g32040* | CGGAAAATCAGATGTTGCGG | GCGCACGTATTCTCTGCCAT | Caldana et al. 2007 |
| *Os12g43530* | GATTTCATCCGACGGATCAAGA | TCACCTTTGGAAGATCCCAGG | Caldana et al. 2007 |
| *Os06g51070* | CATTCCCGACACCATGCAAT | CCTTCCGGTAGACCTTGCAGA | Caldana et al. 2007 |
| *Os09g33490* | TCGTCGTCGAGCTGTGTCA | CCTCTCTTTGAGAGGCAGATGG | Caldana et al. 2007 |
| *Os11g31330* | AAATACCTCTACCCCCGTGCTTT | CAACATCGGTGATGATCGCA | Caldana et al. 2007 |
| *Os02g34970* | TGACATCATCCCGACGCTG | GCTTGCAGTGCTTTGCCATT | Caldana et al. 2007 |
| *Os04g35660* | ATTGAATGGTAAAGCACTCCAATCC | GTCTGCGTCGCATGACTGAA | Caldana et al. 2007 |
| *Os01g59640* | CTGAATCCTCCGAATCTGCGT | CGGCATTGTGCTGCTAATCTG | Caldana et al. 2007 |
| *Os11g04960* | GCAGGCAAATCCCAGATGG | GCGGCCTTTTTATTGCGTTT | Caldana et al. 2007 |
| *No anotated_2* | GGTCGAGATCGGCCTCAAATA | ACACAGAACAAGCTCGTCGGAG | Caldana et al. 2007 |
| *Os10g27390* | AGAACGAGGTTCCGGATCTTG | TCCCTGCCTTCCTTCCTCA | Caldana et al. 2007 |
| *Os03g59730* | GTATCGCATCCCGCAGTTTC | TGTAAGCAGGCGGTTTGAATC | Caldana et al. 2007 |
| *Os06g15690* | TCGGACATCCTGGATAATTGACA | CACCGATCCAGATGCATTTTTT | Caldana et al. 2007 |
| *Os07g13920* | CGATCAGAACCAACAGGATTGG | AGTCATCGCCCATCACACAGA | Caldana et al. 2007 |
| *Os11g31360* | TGTCAACTACCTGTACCGCCGT | TCCACATCGGTGATGAAGTCG | Caldana et al. 2007 |
| *Os01g48130* | TTGCTGACCTGCTGTTGCAT | TGGAAGAAGTGCCGGATCA | Caldana et al. 2007 |
| *Os10g26270* | GGCGACCAAAACCCTGTCA | TTCACCGGCTCTGTGTCCTC | Caldana et al. 2007 |
| *Os10g21560* | GGAATGACCATAACGCAGCTG | CAAACCCTTGTTCAGGTGGACT | Caldana et al. 2007 |
| *Os12g07790* | CGAGATAGTTGGCGCATACGA | CGAAGCCAATGGACAGCATC | Caldana et al. 2007 |
| *Os11g31380* | GAAACAACCATCGCCAAGAGA | ACGGCGAGCACATTACGAA | Caldana et al. 2007 |
| *Os04g40130* | TTACACGGACGAAACGGACCT | AGTGTGATCCCCATCCACCC | Caldana et al. 2007 |
| *Os12g22940* | TCCCAGCTATTTGTTCTGCCC | ACAGCGGTGGATTGCCATT | Caldana et al. 2007 |
| *Os10g27360* | CATCGAGGTTGCGGATCTTG | TGTCCACTCTGGTTCGTGCTC | Caldana et al. 2007 |
| *Os08g10080* | CCTCTCCACCTCAAGGTGAAGACA | CGCAATCCGAACGAAGAACAACAA | Caldana et al. 2007 |
| *Os12g29330* | TCCGACTAAGCTAGGCAAGGCAAG | TTGGGGGCGATTTCGTTAAGCA | Caldana et al. 2007 |
| *No anotated_3* | TTGGACCTGGATGCGTTGCTGT | CGTGTCGAATTCCACTCCCAAACT | Caldana et al. 2007 |
| *Os03g42630* | TGATGCACGAGTACAGGCTCCA | AGCTTCTTGAAGACGCGGCAGA | Caldana et al. 2007 |
| *Os02g36880* | CCGCCAATTCCAAGCAGGATCA | TCGAGGCTCTTCTTGAACACCCT | Caldana et al. 2007 |
| *Os02g36880* | TCATGCACGAGTACCGCATCCA | ACAGCACCCACTCCTGATCCTT | Caldana et al. 2007 |
| *Os04g38720* | TGCTGCAAAAGCAATAAGCAAGGA | TGCTGCTCCATTGCTGCCTAAG | Caldana et al. 2007 |
| *Os10g09820* | CGAGAAATACAAGGGTTACGGAGA | CCATCCACCACCACCTTCCTATT | Caldana et al. 2007 |
| *Os11g08210* | ACAACGCCCTCAGGTTGGATGA | TCGTACCTCTCGATCACTCCCTTC | Caldana et al. 2007 |
| *Os05g34830* | TGGGTCCTGTGCCGAATCTACAAC | GGTCTCCCCGTGGCTCATCATATT | Caldana et al. 2007 |
| *Os05g34830* | AGGAGCTGGTGATGCACTACCT | CGTACAGCGCCATTCTTGTAGAGG | Caldana et al. 2007 |
| *Os06g33940* | GCAAGAAGACCGACTGGATCATGC | CCACCCAACCATCTTCCTGGACAT | Caldana et al. 2007 |
| *Os02g15340* | TGTTTGTGTGCCTTTCCAAGAGTG | TAGAGCAATTCCTCGTCCGTCG | Caldana et al. 2007 |
| *Os11g08210* (*OsNAC5*) | CAGCAGCTGATGGTATTGTC | AGAGACCTGTTTGGCACGAA | Sperotto et al., 2009 |
| *Os01g66120* (*OsNAC6*) | CCAGCCCAAGATCAGCGAGT | TCATGTACTGGGGCAAGCCA | Nakashima et al., 2007 |
| *Os09g35030* (*OsDREB1A*) | GGGATCAAGCAGGAGATGAGCG | TGCCTCGTCTCCCTGAACTTGG | Kim et al., 2010 |
| *Os01g07120* (*OsDREB2A*) | GCTGCACATCAGCACCTTCA | TCCTGCACCTCAGGGACTAC | Matsukura et al. 2010 |
| *Os05g27930* (*OsDREB2B*) | TCCAGCCCGGAAGAAAATGT | GCTCCTGCTGATTGTTGAGC | Matsukura et al. 2010 |
| *Os08g36790* (*TRAB1*) | TCAGGTATTAGAGGCGGTCA | GACAGGGCAAGTTAAGCAC | Yang et al., 2011 |
| *Os09g28310* (*OsbZIP72*) | AATGAGGTAGAAGAAATGAT | GCACAGTCGCTGATGAAGG | Lu et al. 2009 |
| *Os05g49890* (*OsRAN2*) | AGCCAAGCAGGTCACATTCC | AGGTTCGGGTCACCAGCAAG | Zang et al., 2010 |
| *Os11g03300* (*OsNAC10)* | TTCTCCTCGACGGCTCATCC | ATGGATGGCTCAGCAGATTG | Jeong et al., 2010 |
| *Os11g06720 (ASR5)* | CCAGGACGAGTACGAGAGGT | CGATCTCCTCCGTGATCTTG | Arenhart et al., 2016 |
| *Os06g48060 (STAR1)* | TCGCATTGGCTCGCACCCT | TCGTCTTCTTCAGCCGCACGAT | Arenhart et al., 2016 |
| *Os03g50890* (*Actin*) | CTCCCCCATGCTATCCTTCG | TGAATGAGTAACCACGCTCCG | Caldana et al. 2007 |
| *Os05g36290* (*Actin 1*) | ATCCTTGTATGCTAGCGGTCGA | ATCCAACCGGAGGATAGCATG | Caldana et al. 2007 |
| *Os01g59150* (*β-Tubulin*) | GGAGTCACATGCTGCCTAAGGTT | TCACTGCCAGCTTACGGAGG | Caldana et al. 2007 |
| *Os03g08020* (*EF1α*) | GTCATTGGCCACGTCGACTC | TGTTCATCTCAGCGGCTTCC | Caldana et al. 2007 |
