# Supplementary material for: Aluminum Enhances Growth and Sugar Concentration, Alters Macronutrient Status and Regulates the Expression of NAC Transcription Factors in Rice
Source: Front Plant Sci. 2017 Feb 14;8:73. doi: 10.3389/fpls.2017.00073 (PMC5306397; doi:10.3389/fpls.2017.00073)
Supplement: Supplementary file 2 [file Table2.doc]

# Aluminum enhances growth and sugar concentration, alters macronutrient status and regulates the expression of *NAC* transcription factors in rice

Marcos Moreno-Alvarado^1^, Soledad García-Morales^1,2^, Libia Iris Trejo-Téllez^3^, Juan Valente Hidalgo-Contreras^1^, Fernando Carlos Gómez-Merino^1^*

SUPPLEMENTARY MATERIAL

**Supplementary Material S2.** Expression stability (*M*) of selected reference genes used to test the transcription factor gene expression in rice roots. A lower M value indicates more stable expression.

| Locus identifier | Gene name | Expression stability (*M*) | | | |
| --- | --- | --- | --- | --- | --- |
|  |  | R1 | R2 | R3 | Average |
| **Os03g50890** | ***Actin*** | **0.061** | **0.085** | **0.078** | **0.075** |
| Os05g36290 | *Actin I* | 0.080 | 0.101 | 0.087 | 0.089 |
| Os03g08020 | *Elongation factor-1α* | 0.063 | 0.153 | 0.090 | 0.102 |
| Os01g59150 | *β-tubulin* | 0.074 | 0.116 | 0.077 | 0.089 |
