# Supplementary material for: Aluminum Enhances Growth and Sugar Concentration, Alters Macronutrient Status and Regulates the Expression of NAC Transcription Factors in Rice
Source: Front Plant Sci. 2017 Feb 14;8:73. doi: 10.3389/fpls.2017.00073 (PMC5306397; doi:10.3389/fpls.2017.00073)
Supplement: Supplementary file 3 [file Image1.pdf]

Aluminum enhances growth and sugar concentration, alters macronutrient status and regulates the expression of *NAC* transcription factors in rice

Marcos Moreno-Alvarado[1], Soledad García-Morales[1,2], Libia Iris Trejo-Téllez[3], Juan Valente Hidalgo-Contreras[1], Fernando Carlos Gómez-Merino[1]*

SUPPLEMENTARY MATERIAL

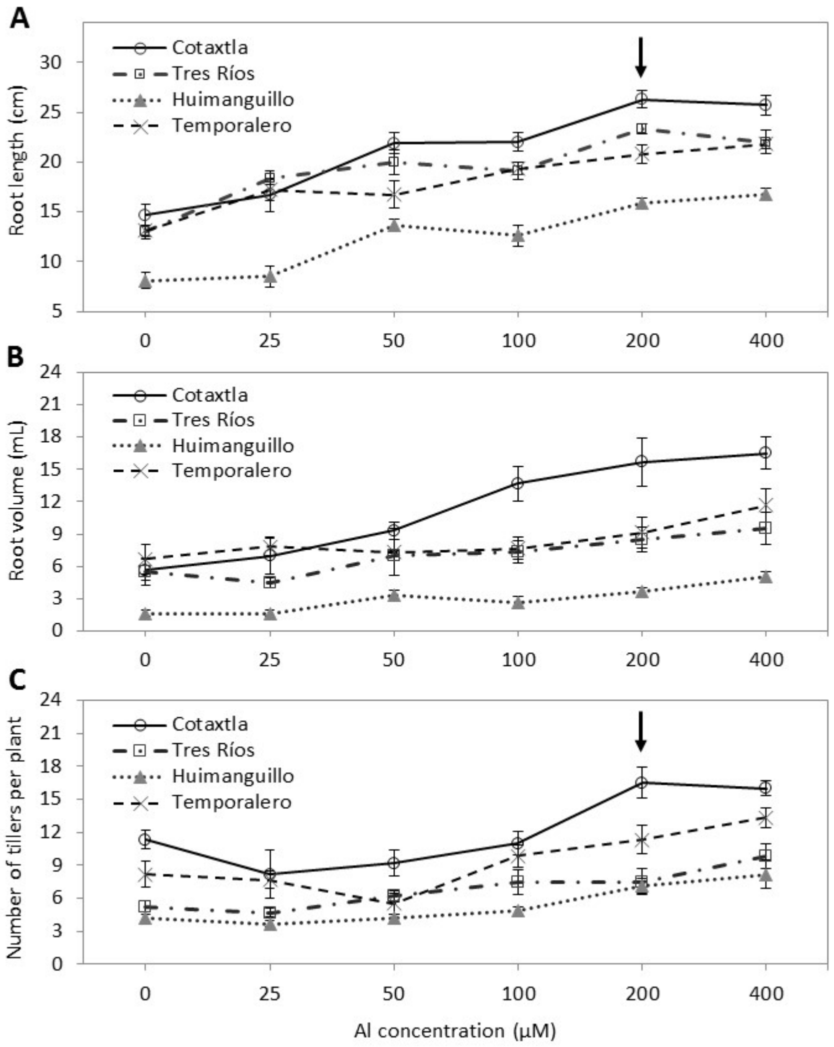

Supplementary Material S3. Root growth and tillers development of rice plants exposed to different Al concentration in the nutrient solution for 25 days. Root length (A), root volume (B), and number of tillers per plant (C) of rice cultivars Cotaxtla, Tres Ríos, Huimanguillo and Temporalero. Rice cultivars were grown with different Al concentrations in hydroponics for 25 days. Values are means ± standard deviation (SD) from at least six individual plants.
